# Performance of established test methods in diagnosing persistent infection at the second stage of a two-stage procedure for periprosthetic hip and knee infections

**DOI:** 10.1302/2633-1462.610.BJO-2025-0159.R1

**Published:** 2025-10-06

**Authors:** Markus Luger, Alexander Bumberger, Constantin Cik, Christoph Böhler, Kevin Staats, Stephan E. Puchner, Reinhard Windhager, Irene Katharina Sigmund

**Affiliations:** 1 Department of Orthopaedics and Trauma Surgery, Medical University of Vienna, Vienna, Austria

**Keywords:** Periprosthetic joint infection, PJI, Diagnosis, Two-stage exchange, Second stage, CRP, White blood cell count, Microbiology, Histology, Sonication, persistent infection, hips, Serum, Synovial fluid, infections, white blood cells, debridement, fibrinogen, antibiotics

## Abstract

**Aims:**

This study aims to evaluate the diagnostic performance of serum parameters, synovial fluid analysis, tissue and sonication fluid cultures, and histology to identify persistent infection, and to predict reinfection at reimplantation of two-stage exchange arthroplasty.

**Methods:**

From January 2015 to January 2023, a total of 133 patients with completed two-stage exchange arthroplasty for periprosthetic joint infection (PJI) following total hip or knee arthroplasty were eligible for inclusion in this retrospective study. Diagnostic values of serum parameters (CRP, white blood cell count (WBC), differential, fibrinogen), synovial fluid WBC (SF-WBC), culture (synovial fluid, tissue, sonication fluid), and histology were evaluated prior to or at the second stage. Additionally, Kaplan-Meier curves were used to determine infection-free prosthesis survival rates for all parameters.

**Results:**

Serum CRP showed the highest area under the receiver operating characteristic curve (AUC; 0.624) among all analyzed test methods (serum WBC: 0.501; serum % polymorphonuclear neutrophils (PMN): 0.605; fibrinogen: 0.533; SF-WBC: 0.601; SF culture: 0.566; tissue culture: 0.463; sonication fluid culture: 0.473; histology: 0.492). Sensitivity, specificity, positive predictive value (PPV), and negative predictive value (NPV) of CRP were 51.7% (95% CI 35 to 69), 73.1% (95% CI 64 to 81), 34.9% (95% CI 21 to 49), and 84.4% (95% CI 77 to 92), respectively. In 35% (n = 15/43) of patients with an elevated serum CRP (≥ 10 mg/l), reinfection occurred, while the reinfection rate was only 16% (n = 14/90) in patients with a normal CRP (< 10 mg/l, p = 0.012). Reinfection rates in patients with all-negative cultures at 23% were not significantly different from cases with positive cultures at 13% (p = 0.352).

**Conclusion:**

Although CRP showed the best diagnostic value among all analyzed test methods, none of them could reliably identify persistent infection or predict reinfection. Additionally, a positive culture may not justify a further intervention (spacer exchange, prolonged antibiotics). In case of positive culture or elevated CRP, a further thorough debridement at the second stage is recommended to increase the chance of infection eradication.

Cite this article: *Bone Jt Open* 2025;6(10):1190–1198.

## Introduction

Two-stage revision arthroplasty is the current ‘gold standard’ procedure for a chronic periprosthetic joint infection (PJI).^1^ However, the diagnosis of a persistent infection before or during the second stage can be difficult. At the moment, no diagnostic test can reliably identify a persistent infection after the first stage of a two-stage revision prior to replantation.^2,3^ Serum parameters such as CRP, synovial fluid parameters like white blood cell count (SF-WBC) and percentage of polymorphonuclear neutrophils (SF-%PMN), and polymorphonuclear neutrophils in histological tissue samples may be elevated due to the inflammatory-immune response after the first procedure, and microorganisms may be difficult to identify due to ongoing antimicrobial therapy.^4-6^

Additionally, no diagnostic test predicting subsequent treatment failure has been found to date. Even patients with all-negative test results at the time of reimplantation may suffer from persistent infection or reinfection.^3,7,8^ Due to this lack of accurate diagnosis, decision-making (further spacer exchange including thorough debridement vs implantation of the definitive prosthesis) can be challenging. However, to improve the outcome (lower reinfection rates and better functional results), surgeons need to know the optimal timing for reimplantation when performing a two-stage revision.^8^

Therefore, the aim of this study was to investigate the diagnostic performance of serum and synovial fluid parameters, microbiology, and histology in detecting persistent infection before or during the second stage (reimplantation) of a two-stage revision arthroplasty for PJI of the hip and knee and in predicting reinfection following two-stage exchange.

## Methods

### Study design

This retrospective analysis of a prospectively collected database was conducted at a single tertiary orthopaedic hospital specialized in treating PJI in accordance with the ethical principles stipulated by the World Medical Association in the Declaration of Helsinki.^9^ Ethical approval was obtained by the institutional ethical review board (EK 2320/2020). From January 2015 to January 2023, patients undergoing revision surgery for PJI of the hip and knee according to the European Bone and Joint Infection Society (EBJIS) definition were identified,^10^ and the surgical procedure was recorded (e.g. debridement, antibiotics, and implant retention (DAIR), one-stage exchange, two-stage exchange). All patients with completed two-stage revision arthroplasty were eligible for inclusion. Patients who did not proceed to replantation of a definitive prosthesis (e.g. Girdlestone (n = 9), amputation (n = 6), spacer as definitive treatment (n = 4)) were excluded. A standardized diagnostic workup was performed prior to the first stage and second stage of a two-stage procedure. At both stages, demographic data, comorbidities, clinical features, and results of serum inflammatory parameters, synovial fluid analysis, microbiology, histology, and sonication were documented. The type of spacer, the spacer interval, spacer exchanges (if performed), and antibiotic treatment prior, during, and after two-stage exchange were recorded. Notably, at our institution patients are under antibiotics without antibiotic holiday before reimplantation.

Reimplantation was undertaken when the general status of the patient was suitable, the wound was healed, soft-tissues were ready for a second surgery, and no clinical or laboratory signs of infection (decreasing inflammatory markers, for example CRP) were present. Patients were reviewed one, three, and 12 months after reimplantation, with further annual follow-up appointments. Patients with a minimum follow-up of 12 months were eligible for accuracy analysis.

### Definition of reinfection

A persistent infection or reinfection was defined as: 1) subsequent revision due to infection with the same initial organism(s) or different organism(s) (DAIR, one-stage exchange, two-stage exchange, resection arthroplasty, amputation, arthrodesis) during the follow-up period after the two-stage revision; 2) use of suppressive antimicrobial therapy due to persistent clinical or laboratory signs of infection; 3) chronic infection without antimicrobial therapy (e.g. chronic sinus tract). Success was defined as the ability to retain the implant after two-stage revision with no clinical or laboratory signs and symptoms of infection, and without the need of suppressive antibiotic therapy.

### Patient demographic characteristics

In total, 133 patients with completed two-stage exchange arthroplasty were included. A reinfection occurred in 29 cases (22%, n = 29/133) after a median period of 13.0 months (IQR 10.0 to 30.0). Regarding age, sex, American Society of Anesthesiologists (ASA) grade,^11^ median spacer interval, and median follow-up time, no difference was observed between the success and failure group (p > 0.05, Table I). Knees (32%, n = 25/78) showed a higher reinfection rate compared to hips (7%, n = 4/55; p = 0.001). A significantly higher BMI was seen in failed cases (p = 0.011).

**Table I.** Demographic characteristics of all included patients, stratified by treatment outcome during the total follow-up period.

Characteristic	Success (n = 104)	Failure (n = 29)	p-value	Total (n = 133)
Median age, years (IQR)	73 (64 to 78)	73 (64 to 77)	> 0.999*	73 (64 to 78)
Female sex, n (%)	57 (54.8)	13 (44.8)	0.341†	70 (52.6)
Median BMI, kg/m^2^ (IQR)	28.1 (24.3 to 32.8)	31.7 (27.7 to 37.2)	0.011*	28.7 (24.8 to 33.7)
ASA grade ≥ III, n (%)	60 (60.6)	16 (57.1)	0.741†	76 (59.8)
**Localization, n (%)**				
Hip	51 (49.0)	4 (13.8)	0.001‡	55 (41.4)
Knee	53 (51.0)	25 (86.2)	0.001‡	78 (58.6)
Median spacer-interval, wks (IQR)	6.6 (5.8 to 7.9)	6.3 (5.1 to 7.3)	0.994*	6.6 (5.7 to 7.9)
Median follow-up time, mths (IQR)	29.5 (24.0 to 44.0)	30.0 (21.0 to 41.0)	0.359*	30.0 (23.0 to 43.0)

* Independent-samples *t*-test.

† Chi-squared test.

‡ Fisher’s exact test.

ASA, American Society of Anesthesiologists.

### Diagnostic tests

Prior to reimplantation, blood samples were taken from all patients. Serum CRP, white blood cell count (WBC), percentage of polymorphonuclear neutrophils (%PMN), and fibrinogen were quantified using a pre-described procedure.^12^ Under sterile conditions, synovial fluid was obtained either before the second stage in the outpatient clinic or in the operating theatre before capsulotomy. SF-WBC was quantified by flow-cytometry (XN-9100; Sysmex, Japan). Furthermore, at least three tissue samples were collected for conventional culture and histopathological analysis. Finally, explanted spacer components were sent for sonication. Cultures (synovial fluid, tissue, and sonication fluid) were incubated on aerobic and anaerobic culture media,^13^ and for histopathological analysis, at least ten high-power fields (HPFs) in each specimen were assessed for neutrophil count as described previously.^14^

For serum CRP, WBC, %PMN, fibrinogen, and SF-WBC, the established cut-off values (≥ 10 mg/l, ≥ 10.10^9^ cells/l, ≥ 70%, ≥ 500 mg/dl, and ≥ 3,000 cells/µl) were used.^12,15^ Synovial fluid culture was considered positive if microbial growth was detected in at least one aspirate. Tissue culture was considered positive when at least two tissue samples showed microbial growth. Sonication fluid culture was considered positive when the colony count exceeded 50 colony forming units (CFU)/ml. Histology was defined positive when ≥ five PMNs in ≥ ten HPFs were seen.

### Statistical analysis

Continuous variables are given as median and IQRs, categorial variables are described as absolute and relative frequencies (percentage). Independent-samples *t*-test, chi-squared test, and Fisher’s exact test were used to compare metric and binary variables as appropriate. For performance analysis, sensitivity, specificity, accuracy, positive (PPV) and negative predictive value (NPV), positive (LR+) and negative likelihood ratio (LR-), and area under the receiver operating characteristic (ROC) curves with their respective 95% CIs were calculated for all diagnostic parameters. Area under the receiver operating characteristic curves (AUC) of all parameters wer compared using the z-test. Additionally, optimal cut-off values were calculated using the Youden index (with priority on specificity).^16^ Kaplan-Meier analysis and log-rank test were used to compare infection-free survival rates over time for each of the analyzed tests. XLSTAT version 2023.1.2.1406 (Lumivero, USA) was used for statistical analysis. Statistical significance was set at p < 0.05.

## Results

The causing microorganisms at first and second stage and reinfection/persistent infection are given in Table II. In five of the reinfected cases (17.2%, n = 5/29), the identical microorganism was observed compared to the index infection (*Staphylococcus aureus* (n = 4) and Coagulase-negative staphylococci (n = 1)). Four culture-negative infections (9.3%, n = 4/43) remained culture-negative at reinfection.

**Table II.** Distribution of cultured microorganisms.

Microorganism	First stage (n = 133), n (%)	Positive at second stage, n (%)	Causing microorganism (second stage)	Reinfection/persistent infection (n = 29), n (%)	Causing microorganisms (reinfection/persistent infection)
*Staphylococcus aureus*	26 (19.5)	2/26 (7.7)	*Corynebacterium* spp., *S. aureus*	9/26 (34.6)	*S. aureus* (4×), *E. coli*, *E. faecium*, *Streptococcus* spp., CN, polymicrobial
CoNS	25 (18.8)	6/25 (24.0)	CoNS (3×), *E. faecalis*, *Bacillus cereus*, *Acinetobacter baumanii*	2/25 (8.0)	CoNS, CN
Streptococci	7 (5.3)	0/7 (0.0)	-	2/7 (28.6)	CoNS, CN
Cutibacterium spp.	5 (3.8)	1/5 (20)	CoNS	0/5 (0.0)	-
Enterobacteriaceae	4 (3.0)	0/4 (0.0)	-	2/4 (50)	2× CN
*Candida albicans*	1 (0.8)	0/1 (0.0)	-	0/1 (0.0)	-
Others*	10 (7.5)	1/10 (10.0)	CoNS	2/10 (20.0)	*Streptococcus* spp. (2×)
Polymicrobial	12 (9.0)	0 (0.0)	-	2/12 (16.7)	CoNS, *Candida albicans*
CN	43 (32.3)	6 (14.0)	CoNS (2 x), polymicrobial, *Steptococcus* spp., *Aspergillus* spp., *Veillonella atypica*	10/43 (23.3)	CN (4×), CoNS (2×), *S. aureus*, *Candida albicans*, *Corynebacterium* spp., *E. coli*

* Others: *Finegoldia magna*, Enterococcus spp. (n = 3)*, Mycobacterium *spp., *Corynebacterium aurimucosum, *Paenibacillus spp., *Pseudomonas aeruginosa*, *Parvimonas micra*, *Listeria monocytogenes, Pasteurella multocida, Veillonella atypica, Bacillus cereus, Acinetobacter baumanii.*

CN, culture-negative; CoNS, coagulase-negative staphylococci.

Diagnostic accuracies of all serum parameters (serum CRP, serum WBC, serum %PMN, and serum fibrinogen) are shown in Table III. The calculated cut-off values did not outperform the predefined values.

**Table III.** Diagnostic accuracies of serum parameters in predicting reinfection. Values are reported as % (95% CI).

Diagnostic accuracy	Cut-off	Sensitivity	Specificity	Accuracy	PPV	NPV	LR+	LR-	AUC	p-value*
Serum CRP (established)	≥ 10 mg/l	51.7 (34.5 to 68.6)	73.1 (63.8 to 80.7)	68.4 (60.5 to 76.3)	34.9 (20.6 to 49.1)	84.4 (77.0 to 91.9)	1.921 (1.197 to 3.084)	0.661 (0.445 to 0.980)	0.624 (0.522 to 0.726)	0.864
Serum CRP (calculated)	≥ 10.6 mg/l	51.7 (34.5 to 68.6)	74.0 (64.8 to 81.5)	69.2 (61.3 to 77.0)	35.7 (21.2 to 50.2)	84.6 (77.2 to 92.0)	1.992 (1.235 to 3.215)	0.652 (0.440 to 0.966)	0.629 (0.527 to 0.731)	
Serum WBC (established)	≥ 10×10^9^ cells/l	6.9 (1.0 to 23.3)	93.3 (86.5 to 96.9)	74.4 (67.0 to 81.8)	22.2 (0.0 to 49.4)	78.2 (71.0 to 85.5)	1.025 (0.225 to 4.669)	0.998 (0.893 to 1.116)	0.501 (0.448 to 0.554)	0.333
Serum WBC (calculated)	≥ 10.6×10^9^ cells/l	6.9 (1.0 to 23.3)	97.1 (91.4 to 99.3)	77.4 (70.3 to 84.5)	40.0 (0.0 to 82.9)	78.9 (71.8 to 86.0)	2.391 (0.419 to 13.638)	0.959 (0.864 to 1.064)	0.520 (0.470 to 0.570)	
Serum %PMN (established)	≥ 70%	37.5 (21.2 to 57.4)	83.5 (73.6 to 90.2)	72.8 (64.2 to 81.4)	40.9 (20.4 to 61.5)	81.5 (73.0 to 89.9)	2.279 (1.113 to 4.666)	0.748 (0.541 to 1.035)	0.605 (0.498 to 0.712)	0.737
Serum %PMN (calculated)	≥ 71.2%	33.3 (18.0 to 53.5)	89.9 (80.9 to 95.0)	76.7 (68.5 to 84.9)	50.0 (25.5 to 74.5)	81.6 (73.5 to 89.7)	3.292 (1.383 to 7.833)	0.742 (0.554 to 0.994)	0.616 (0.514 to 0.718)	
Serum fibrinogen (established)	≥ 500 mg/dl	17.2 (7.3 to 35.2)	89.3 (81.7 to 94.0)	73.5 (66.0 to 81.0)	31.3 (8.5 to 54.0)	79.3 (71.9 to 86.7)	1.614 (0.610 to 4.274)	0.927 (0.775 to 1.108)	0.533 (0.457 to 0.609)	0.157
Serum fibrinogen (calculated)	≥ 529 mg/dl	17.2 (7.3 to 35.2)	96.1 (90.0 to 98.8)	78.8 (71.8 to 85.8)	55.6 (23.1 to 88.0)	80.5 (73.5 to 87.5)	4.440 (1.274 to 15.474)	0.861 (0.726 to 1.021)	0.567 (0.494 to 0.639)	

* z-test.

AUC, area under the curve; LR-, negative likelihood ratio; LR+, positive likelihood ratio; NPV, negative predictive value; %PMN, percentage of polymorphonuclear neutrophils; PPV, positive predictive value; WBC, white blood cells.

Accuracies of other diagnostic tests (SF-WBC, SF culture, tissue and sonication culture, as well as histology) are summarized in Table IV.

**Table IV.** Diagnostic accuracy of diagnostic tests in predicting reinfection at the one-year follow-up. Values are presented as % (95% CI).

Diagnostic accuracy	Cut-off	Sensitivity	Specificity	Accuracy	PPV	NPV	LR+	LR-	AUC
SF-WBC	≥ 3,000 cells/µl	27.3 (9.5 to 57.2)	92.9 (76.0 to 99.0)	74.4 (60.7 to 88.1)	60.0 (17.1 to 100)	76.5 (62.2 to 90.7)	3.818 (0.735 to 19.835)	0.783 (0.538 to 1.141)	0.601 (0.454 to 0.747)
≥ 1 positive culture*	≥ 1 positive culture	7.4 (1.1 to 24.7)	85.9 (77.5 to 91.5)	69.0 (61.0 to 77.1)	12.5 (0.0 to 28.7)	77.3 (69.4 to 85.1)	0.524 (0.127 to 2.165)	1.078 (0.944 to 1.232)	0.466 (0.405 to 0.527)
SF culture	≥ 1 positive culture	16.7 (3.8 to 46.2)	96.6 (81.1 to 100)	73.2 (59.6 to 86.7)	66.7 (13.3 to 100)	73.7 (59.7 to 87.7)	4.833 (0.483 to 48.414)	0.863 (0.664 to 1.122)	0.566 (0.451 to 0.681)
Tissue culture	≥ 2 positive cultures with the same microorganism	4.0 (0.0 to 21.4)	88.6 (79.5 to 94.0)	68.3 (59.3 to 77.2)	10.0 (0.0 to 28.6)	74.5 (65.7 to 83.3)	0.351 (0.047 to 2.637)	1.083 (0.968 to 1.212)	0.463 (0.410 to 0.516)
Sonication culture	> 50 CFU/ml	0.0 (0.0 to 21.1)	94.7 (86.6 to 98.2)	76.3 (67.7 to 85.0)	0.0 (0.0 to 0.0)	79.8 (71.4 to 88.1)	0.0 (-)†	1.056 (1.001 to 1.115)	0.473 (0.448 to 0.499)
Histology	≥ 5 PMNs in ≥ 10 HPFs	37.9 (22.7 to 56.1)	60.4 (50.6 to 69.4)	55.4 (46.8 to 63.9)	21.6 (10.3 to 32.9)	77.2 (68.0 to 86.5)	0.958 (0.567 to 1.618)	1.028 (0.742 to 1.423)	0.492 (0.390 to 0.593)

* At least one single positive culture in either synovial fluid, tissue, or sonication fluid.

† Due to sensitivities of 0%, LR+ was calculated to be 0, and (per definition) no useful CI can be given.

AUC, area under the curve; CFU, colony forming unit; HPF, high-power field; LR+, positive likelihood ratio; LR-, negative likelihood ratio; NPV, negative predictive value; PMN, percentage of polymorphonuclear neutrophils; PPV, positive predictive value; SF, synovial fluid; WBC, white blood cells.

**Fig. 1:**
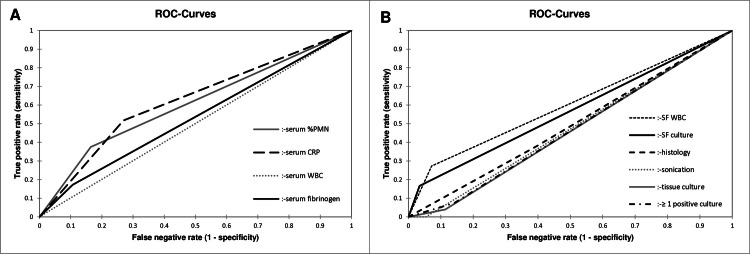
Receiver operating characteristic (ROC) curves for accuracy of a) serum parameters and b) ≥ one positive culture, histology, synovial fluid white blood cells (WBC), synovial fluid culture, tissue culture, and sonication fluid culture in detecting reinfection or persistent infection at the one-year follow-up. %PMN, percentage of polymorphonuclear neutrophils; SF-culture, synovial fluid culture; SF-WBC, synovial fluid white blood cell count.

Among all diagnostic tests, serum CRP (AUC 0.624; 95% CI 0.522 to 0.726), serum %PMN (0.605; 95% CI 0.498 to 0.712), and SF-WBC (0.601; 95% CI 0.454 to 0.747) showed the best performances at reimplantation. Figure 1 shows ROC curves of serum parameters, synovial fluid analysis, microbiology, and histology. All p-values (independent-samples z-test) for differences in AUCs are shown in Table V. No statistically significant differences in diagnostic accuracies (AUC) were found when analyzing hip and knee cases separately. When evaluating all possible pairwise combinations of the investigated parameters (where a combination was considered positive if at least one of the parameters yielded a positive result), no combination demonstrated a statistically significant improvement in diagnostic performance compared to CRP alone (p > 0.05).

**Table V.** p-values of the independent-samples z-test comparing the area under the receiver operating characteristic curve of all parameters.

Parameter	Serum CRP	Serum fibrinogen	Serum %PMN	Serum WBC	SF-WBC	≥ 1 positive culture	SF culture	Tissue culture	Sonication culture	Histology
Serum CRP*	1	0.001	0.537	< 0.001	0.629	< 0.001	0.180	< 0.001	< 0.001	< 0.001
Serum fibrinogen*	0.001	1	0.012	0.153	0.155	0.005	0.427	0.003	0.005	0.119
Serum %PMN*	0.537	0.012	1	< 0.001	0.927	< 0.001	0.380	< 0.001	< 0.001	< 0.001
Serum WBC*	< 0.001	0.153	< 0.001	1	0.035	0.105	0.113	0.080	0.139	0.708
SF-WBC*	0.629	0.155	0.927	0.035	1	0.006	0.552	0.005	0.007	0.027
≥ 1 positive culture	< 0.001	0.005	< 0.001	0.105	0.006	1	0.018	0.884	0.722	0.321
SF culture	0.180	0.427	0.380	0.113	0.552	0.018	1	0.015	0.024	0.087
Tissue culture	< 0.001	0.003	< 0.001	0.080	0.005	0.884	0.015	1	0.607	0.266
Sonication culture	< 0.001	0.005	< 0.001	0.139	0.007	0.722	0.024	0.607	1	0.432
Histology	< 0.001	0.119	< 0.001	0.708	0.027	0.321	0.087	0.266	0.432	1

* The established cut-off values were used for the calculations.

%PMN, percentage of polymorphonuclear neutrophils; SF, synovial fluid; WBC, white blood cells.

**Fig. 2:**
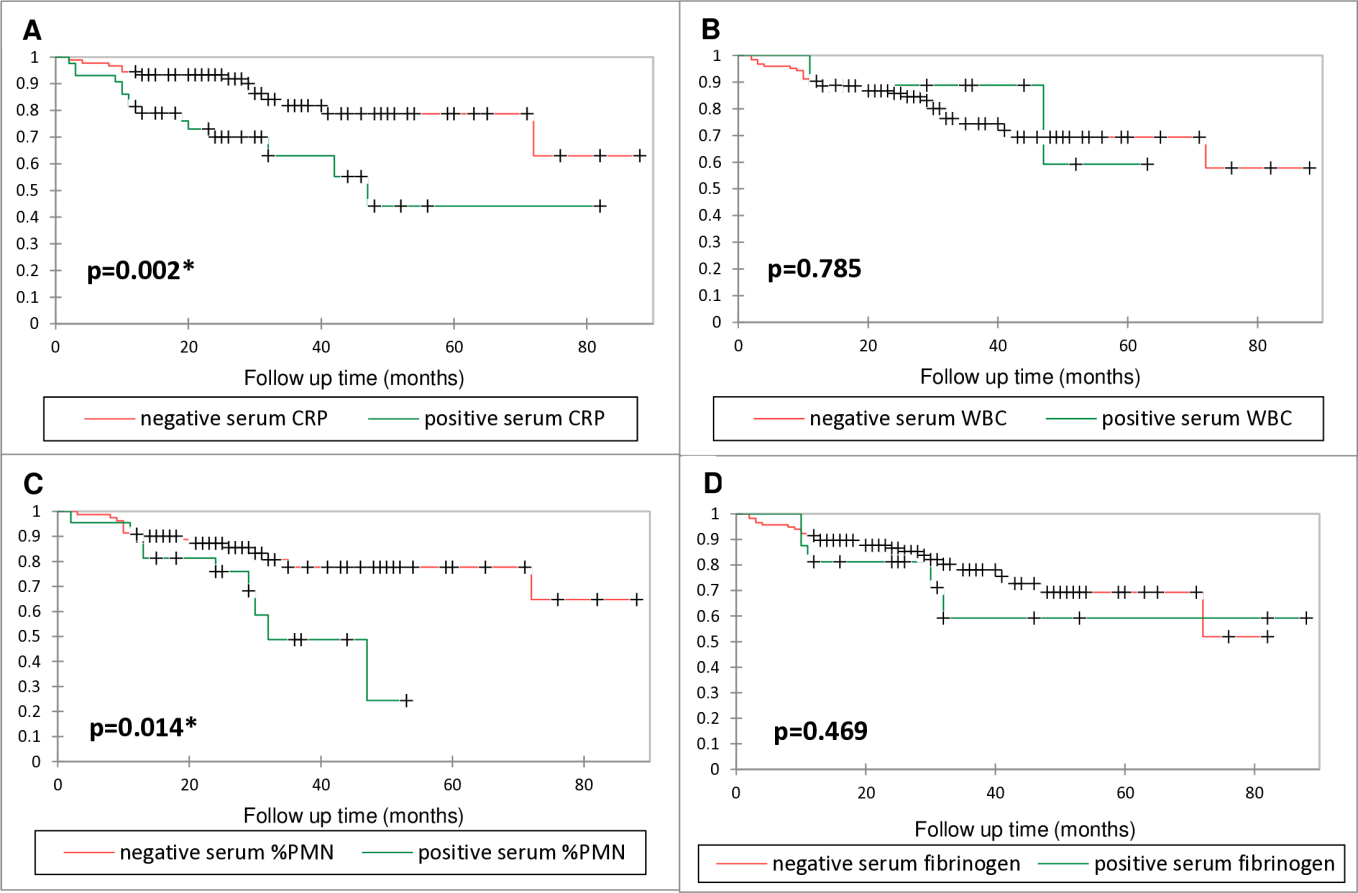
Kaplan-Meier graphs showing infection-free survival of patients with elevated or normal serum parameter levels at reimplantation. a) CRP. b) White blood cell count (WBC). c) Percentage of polymorphonuclear neutrophils (PMN). d) Fibrinogen. + indicates censored data; *indicates significance at p < 0.05 (log-rank test).

Kaplan-Meier curves regarding infection-free survival over time for serum parameters are shown in Figure 2. In cases with positive serum CRP (≥ 10 mg/l, range: 10.6 to 83.5 mg/l), the reinfection rate was 35% (n = 15/43), and 16% (n = 14/90) in the negative serum CRP group (< 10 mg/l; p = 0.012, chi-squared test). The infection-free survival probability was 91% (95% CI 82 to 99) in the positive CRP group and 97% (95% CI 93 to 100) in the negative serum CRP group at six months. Survival probabilities in the positive and negative CRP groups were 81% (95% CI 70 to 93) and 94% (95% CI 90 to 99) at 12 months, and 70% (95% CI 56 to 85) and 93% (95% CI 88 to 99) at two years, respectively. The mean infection-free survival times in the positive and negative serum CRP groups were 37 months (95% CI 31 to 43) and 68 months (95% CI 62 to 75), respectively. A higher infection-free survival rate was observed in CRP-negative cases (p = 0.002). In cases with negative serum %PMN (< 70%) significantly longer infection-free survival times were seen (p = 0.014). At two years, the infection-free survival probability in the negative serum %PMN group (< 70%) was 87% (95% CI 80 to 95), and 76% (95% CI 57 to 94) in the positive serum %PMN group (≥ 70%).

**Fig. 3:**
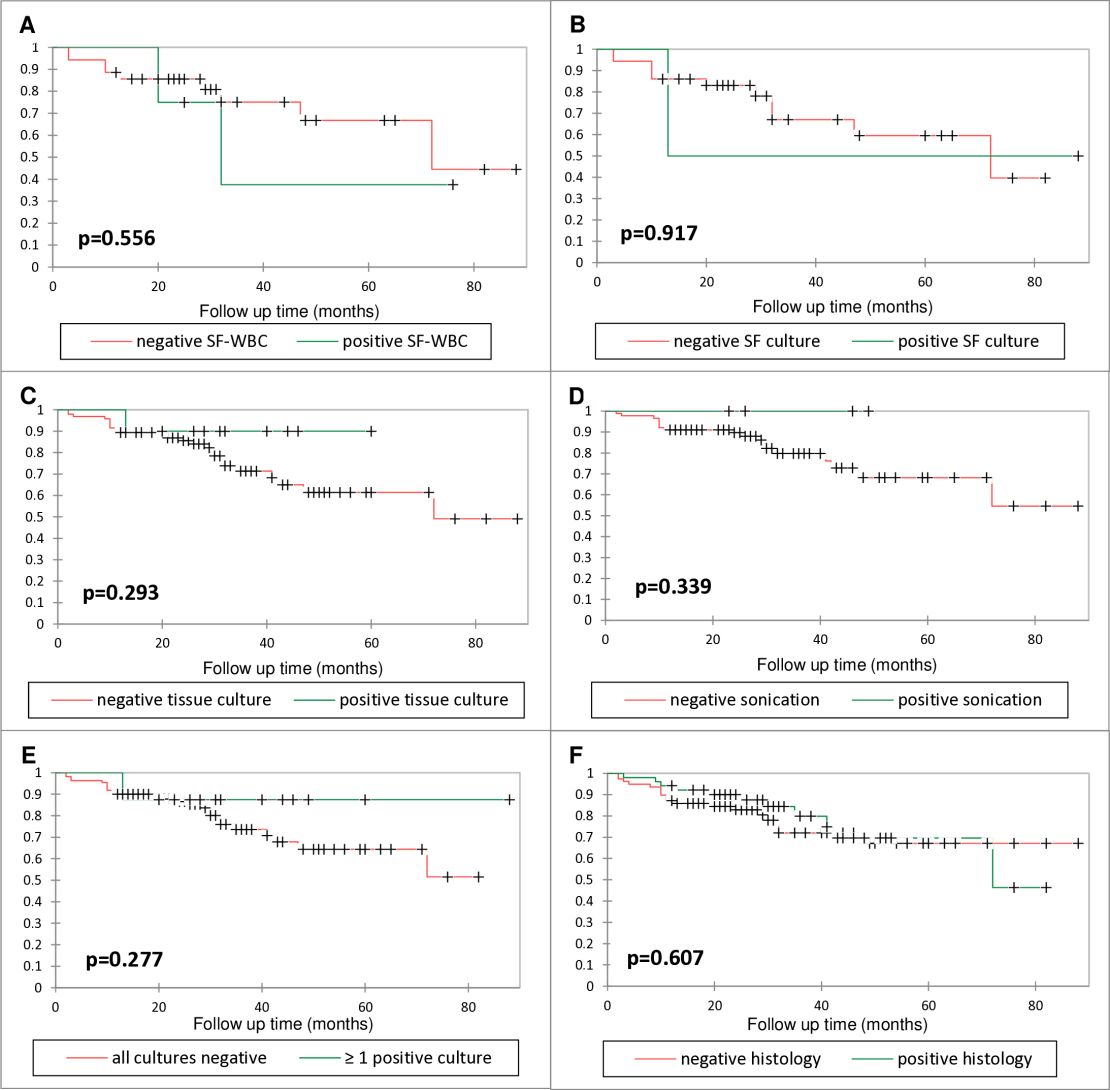
Kaplan-Meier graphs showing infection-free survival of cases with a) elevated and normal synovial fluid white blood cell count (SF-WBC), b) positive and negative synovial fluid culture, c) tissue culture, d) sonication fluid culture, e) ≥ one positive culture (synovial fluid, tissue, or sonication), and f) histology at reimplantation. + indicates censored data.

Figure 3 summarizes infection-free survival over time for SF-WBC, ≥ one positive culture, SF-culture, tissue culture, sonication, and histology. No statistically significant difference between Kaplan-Meier curves for any of the parameters was observed. The graphs for tissue culture, sonication culture and ≥ one positive culture display an inverse relationship regarding survival rates. For tissue culture, only one failure was recorded for cases with a positive result.

## Discussion

Serum CRP showed the highest accuracy among all analyzed diagnostic test methods to predict persistent infection or reinfection in our cohort. In 35% of patients with an elevated serum CRP (≥ 10 mg/l) prior to replantation, a persistent or reinfection occurred, while the reinfection rate was only 16% in patients with a normal CRP (< 10 mg/l, p = 0.012). Furthermore, Kaplan-Meier analysis showed lower infection-free survival rates in patients with elevated CRP levels (p = 0.002), suggesting improved outcomes in patients with normal (< 10 mg/l) CRP at reimplantation. This parameter along clinical signs and general status of patients is also the most widely used marker among orthopaedic surgeons prior to reimplantation for decision making (reimplantation vs spacer exchange).^17^ However, the overall accuracy of CRP was only moderate in our cohort. The high false-negative rate may be explained by the ongoing antimicrobial therapy during the prosthesis-free interval suppressing bacterial growth and/or the reduced systemic immune response in PJIs caused by low-virulence organisms. Hence, a normal serum CRP cannot exclude a persistent infection. In addition, an elevated CRP prior to replantation can actually reflect the continued infection, but the new thorough debridement during the second stage can lead to eradication of the remaining microorganisms.^18^ Furthermore, the high false-positive rate may also be explained by the inflammatory-immune response after the first procedure and/or another (still existing) infectious focus at a site other than the affected joint.^2^ Therefore, an elevated serum CRP cannot accurately predict a persistent or reinfection after two-stage exchange. Similar low accuracies of CRP were reported in the literature, with two studies being similar to ours in methodology. Pannu et al^19^ reported a sensitivity of 22% and specificity of 73% in their retrospective analysis of 44 reimplantation cases (17 hips, 27 knees), using the Delphi consensus for reinfection and a minimum follow-up of one year. Li et al^20^ conducted a retrospective study of 130 two-stage revisions (57 knees, 73 hips) and a follow-up of two years. CRP showed a sensitivity of 82% and a specificity of 50% in predicting infection eradication. In a meta-analysis by Khan et al^2^ including eight studies compromised of 611 patients, the pooled sensitivity and specificity for CRP assessing failure after replantation were 45% (95% CI 35 to 54) and 72% (95% CI 68 to 76). However, frequently used Delphi criteria for treatment success after reimplantation do not differentiate between revision surgeries for septic and aseptic causes,^21^ whereas we did not consider revisions for aseptic causes (e.g. loosening, wear, etc) as reinfections.

To the best of our knowledge, this is the first study evaluating serum %PMN for the prediction of reinfection. Although its immediate diagnostic performance was low with an AUC of 0.605 (95% CI 0.498 to 0.712), Kaplan-Meier analysis revealed a significantly lower survival rate for serum %PMN-positive cases (p = 0.014). A total of nine of 21 (31%) failed cases had elevated serum %PMN, compared with 13/104 cases (13%) in the successful group (p = 0.028). Outcome may be worse in elevated serum %PMN cases, but further studies are needed to build upon these findings.

Overall, cultures showed good specificities (synovial fluid 97%, tissue 89%, and sonicate fluid 95%), but failed to identify most of the reinfection cases in our series (sensitivity: synovial fluid 17%, tissue 4%, and sonicate fluid 0%). Reinfection rates were not significantly different between patients with all-negative cultures (23%, n = 25/110) and patients with positive cultures (12%, n = two/16; p = 0.352, ≥ one positive culture obtained from synovial fluid, tissue, or sonicate fluid). As mentioned above, a possible explanation of the high false-negative rate may be the ongoing antimicrobial therapy during the prosthesis-free interval without honeymoon period. Furthermore, a positive culture prior to or during the second stage does not necessarily lead to a higher reinfection rate after the second stage.^22-26^ The microorganism can still be present, but the additional thorough debridement during the second stage can eradicate the still existing microorganisms. Therefore, positive cultures may not justify further surgical (spacer exchange) or antibiotic treatment delaying reimplantation until cultures are negative.

While specificities of serum WBC, serum fibrinogen, and synovial fluid WBC were good, sensitivities were poor. Additionally, histology showed an overall poor performance. Hence, none of these parameters can be recommended for the identification of a persistent infection at the second stage of a two-stage revision.

To our knowledge, this is the first study evaluating the performance of serum parameters, synovial fluid tests, culture, and histology in a single patient cohort. However, this study has limitations. Not all parameters were always available for all patients, which reflects clinical practice. Our study did not include analysis of synovial fluid neutrophil percentage, as the low absolute leucocyte counts in many samples (< 1.0 G/l) prevented differential analysis using our institution’s automated system, despite this marker being recognized as highly sensitive for diagnosing PJI. Additionally, reinfections may occur after our follow-up period. Due to our analysis of only revision cases with replantation (without resection arthroplasty, arthrodesis, amputation, etc), some of our calculations should be interpreted with caution. Furthermore, it is often difficult to differentiate between a persistent infection (reinfection caused by the same microorganism present at first or second stage) and a reinfection (reinfection caused by a different microorganism compared to the first or second stage).^27,28^ Indeed, in our cohort, only five of the 29 reinfections (17%) showed growth of the identical microorganism(s), while in the remaining 24 (83%) patients, a different microorganism was cultured. Hence, it is possible that some of the infections have been cured by the two-stage exchange (+ antimicrobial therapy) and the patient experienced a new PJI caused by another microorganism.^29,30^ However, it can also be that the ‘newly’ cultured microorganism may have been present all along but has not been identified during the first or second stage. Hence, the accurate definition of persistent infection and reinfection is difficult.

In conclusion, serum CRP showed the best diagnostic value among all analyzed test methods to predict persistent infection or reinfection at the second stage. One-third of patients with an elevated serum CRP (≥ 10 mg/l) prior to replantation experienced a reinfection. However, the overall accuracy of all investigated test methods (including CRP) was insufficient to reliably predict persistent infection/reinfection in our cohort. Additionally, patients with positive cultures at the second stage had no higher reinfection rate after two-stage exchange, hence, a positive culture may not justify a further surgical intervention (spacer exchange) or prolonged antimicrobial therapy. However, in case of positive culture or elevated CRP, a further thorough debridement should be performed at the second stage to increase the chance of infection eradication.

**Take home message**

- Patients with an elevated serum CRP (≥ 10 mg/l) prior to replantation had a higher risk of reinfection.

- Although serum CRP demonstrated the highest diagnostic value among all investigated test methods, its overall accuracy was still insufficient to reliably predict persistent infection or reinfection at the second stage.

- A positive culture at the second stage was not associated with higher reinfection rates, and may not justify additional surgery or prolonged antibiotic therapy.

## Data Availability

All data generated or analyzed during this study are included in the published article.
